# Effect of Chia as Breadmaking Ingredient on Nutritional Quality, Mineral Availability, and Glycemic Index of Bread

**DOI:** 10.3390/foods9050663

**Published:** 2020-05-20

**Authors:** Karla Miranda-Ramos, Ma. Carmen Millán-Linares, Claudia Monika Haros

**Affiliations:** 1 Institute of Agrochemistry and Food Technology (IATA-CSIC), 46980 Valencia, Spain; karla.mirandara@ug.edu.ec; 2 Faculty of Chemical Engineering, University of Guayaquil, Cdla. Universitaria Salvador Allende Malecón del Salado entre Av. Delta y Av. Kennedy, 090510 Guayaquil, Ecuador; 3 Vegetable Protein Group, Instituto de la Grasa (IG-CSIC), 41013 Seville, Spain; mcmillan@ig.csic.es

**Keywords:** *Salvia hispanica*, chia ingredients, breadmaking products, fatty acid profile, essential amino acid profile, minerals, PRI/RDA/AI (Popular Reference Intake/Recommended Dietary Allowance/Adequate Intake)

## Abstract

Chia seeds and chia flour could be used as ingredients to enrich foods owing to their high amount of nutrients. The goal of this investigation was to provide further information about how replacing wheat flour with chia ingredients (seeds, whole flour, semi-defatted, and low-fat flours) affects the nutritional and functional value of bread. The higher levels of proteins, lipids and minerals determined in raw chia flours directly affected the increase of these nutrients. High levels of phytates were found in chia ingredients (5.1–6.6 μmol/g d.b.), which affect Zn and Fe bioavailability, as predicted by phytate/mineral molar ratios. Bread with chia had a high amount of linoleic acid, especially in bread with chia seeds, owing to protection of seed integrity during baking. Chia ingredients did not show limiting essential amino acids such as lysine, which is deficient in cereals. Glycemic index was lower in bread with chia ingredients compared to control. The beneficial effects on glucose metabolism together with the nutritional and functional characteristics could be clinically important for prevention of metabolic diseases.

## 1. Introduction

The increasing consumer demand for nutritious, healthy food has made the food industries examine their own resources to discover and take advantage of functional ingredients. Nowadays, with regard to bakery goods, wheat bread has been enriched with new food ingredients [1,2]. Numerous epidemiological and experimental studies suggest that changes in the diet are decisive in the prevention of various metabolic disorders included in the so-called metabolic syndrome, such as type 2 diabetes, insulin resistance, hypertension, obesity, and cardiovascular disease [3]. Furthermore, intake of prebiotic foods, wholegrain, high fiber, seed breads, or high amounts of omega-3 leads to lower blood cholesterol and consequently reduces the risk of cardiovascular disease [4].

The seeds of *Salvia hispanica* (chia) have high nutritional and functional characteristics. Its oil has predominantly unsaturated fatty acids, such as α-linolenic acid (64.4%) and linoleic acid (21.5%), and less than 10% of saturated fatty acids [5]. Chia seed oil has a low n-6/n-3 ratio, therefore intake of it could help to get the ratio between 5:1 and 9:1, in accordance with WHO/FAO (World Health Organization/Food and Agriculture Organization) [6] and EFSA (European Food Safety Authority Food) Panel on Dietetic Products and Allergies [7] recommendations to prevent the development of metabolic disorders, tumor cells, and chronic diseases [8]. The seeds are also a more abundant source of proteins (19–27%) than conventional crops such as rice (5.95%), oat (13.15%), and wheat (9.61%) [9,10]. Chia proteins contain high concentrations of essential amino acids such as lysine, leucine, isoleucine, and valine [11]. These proteins have a complete amino acid profile, unlike cereals, which are particularly deficient in lysine in comparison with the scoring pattern for children (1–2-years-old) which is taken as a reference [12,13]. In addition, the seeds have a high amount of fiber (18–40%), more than other grains such as cereals and legumes, and it is mainly soluble [3,14]. Soluble fiber (gums, pectins, and mucilages) has bioactive effects, such as enhancing the immune function, lowering cholesterol and delaying starch digestion and glucose release from foods, with a consequent decrease in post-prandial glycaemia [15]. Furthermore, the mucilage of chia seeds could be linked to starch in bread baking products, impeding starch gelatinization and thus enzymatic vulnerability, and lowering the glycemic index [15,16]. Moreover, it has been found that chia seeds contain a high number of phenolic compounds and high concentrations of natural antioxidants, such as quercetin and kaempferol, while caffeic and chlorogenic acids are present in low concentrations [17]. Chia can be considered a seed with antioxidative potential and could be used as an antihypertensive substance [18]. Chia has a high concentration of minerals, but the bioavailability of di- or trivalent cations, such as calcium, iron, or zinc, depends on the phytate concentration, which may decrease during food processing [19].

Because of its high nutritional properties, consumption of chia has spread widely in the European Union (EU), and in the EU list of novel foods EFSA has authorized the use of chia seeds up to maximum inclusion levels [20]. Whole chia seeds may be marketed in the European community as a food ingredient for use in baked products and breakfast cereals up to 10%; ground chia seeds up to 5% in bread; whole chia seeds up to 5% in sterilized ready-to-eat meals based on cereal/pseudo-cereal grains and/or pulses; pre-packaged chia seed as such, and fruit/nut/seed mixes; and chia in confectionery products and chocolates; edible ices; fruit and vegetable products; non-alcoholic beverages and puddings (<120 °C in their preparation) without limit, according to the European Commission [21]. Recently, the use of two partially defatted powders of chia enriched with proteins or fibers was authorized as food supplements for the adult population (up to 7.5 and 12 g/day, respectively), or as nutritional ingredients in a variety of foods (yogurt, vegetable beverages, energy drinks, chocolate, fruit, and pasta) at a level of 0.7–10% [22]. The partial replacement of wheat by chia seeds, whole chia flour and defatted chia flour in bread (up to a level of 5–6%) obtained high consumer acceptance in earlier studies, and it could extend the shelf life of bread, since it inhibits the kinetics of retrogradation of amylopectin during storage [2,16]. However, the breadmaking process may affect the stability and/or bioavailability of nutrients/bioactive compounds owing to various chemical and enzymatic reactions during kneading, fermentation, and baking, and accordingly this enriched bread would provide a greater or lesser health benefit [23].

The nutritional value of food depends on many factors, but mainly on the amounts of macronutrients (proteins, fats, carbohydrates) and micronutrients (minerals and vitamins). The lack or excess of some of these substances can have detrimental effects on health, and therefore the EFSA [24] and FAO/WHO/UNU (United Nations University) [13] have developed and applied dietary reference intakes (DRIs), which are the minimum amount of a particular nutrient that can be consumed daily without health risks in order to maintain the health and well-being of the body.

The objective of this study was to characterize and analyze the potential of chia seeds, whole chia flour, and the defatted chia flour obtained after extraction of chia oil (currently considered as waste in the EU) as nutritional and functional bakery ingredients. Further aims were to study the effect of baking on the amino acid and fatty acid profiles, mineral availability and the contribution to nutrient DRIs, and to estimate the glycemic index of the bakery products developed.

## 2. Materials and Methods

### 2.1. Materials

Commercial Spanish wheat flour (W) was purchased from La Meta (Lleida, Spain). Chia seeds (CWS), chia whole flour (CWF), semi-defatted chia flour (CSDF), and low-fat chia flour (CLFF) were donated by Chia S.A. Company (Valencia, Spain). CSDF and CLFF were obtained by supercritical CO_2_ extraction. The characteristics of wheat flour (W) and chia ingredients were described in an earlier study by Iglesias-Puig and Haros [16]. Compressed yeast (*Saccharomyces cerevisiae*; Levamax, Valencia, Spain) was used as a starter for the breadmaking process.

### 2.2. Breadmaking Process

The control bread dough formula consisted of wheat flour (500 g), compressed yeast (2.5% flour basis), sodium salt (1.6% flour basis) and distilled water (up to optimum absorption, 500 Brabender Units). The ingredients were mixed for 4 min, rested for 10 min, divided (100 g), kneaded and then rested (15 min). The breadmaking process was carried out according to the method previously described by Iglesias-Puig and Haros [16]. The various bread products studied were control bread (WB); whole seed bread (CWSB5 and CWSB10); whole flour bread (CWFB5 and CWFB10); semi-defatted flour bread (CSDFB5 and CSDFB10) and low-fat chia flour bread (CLFFB5 and CLFFB10), where 5 and 10 mean with 5% and 10% of chia ingredient on flour basis.

### 2.3. Composition of Raw Materials and Breads

Moisture was determined by a method of the AACC (American Association of Cereal Chemists) [25], ash content was determined in a muffle by incineration at 910 °C [26], and protein was quantified by the Kjeldahl method of the AACC [27]. Lipid content was extracted with hexane or petroleum ether reflux conditions by the Soxhlet technique, and dietary fiber content was measured by the soluble, insoluble, and total dietary fiber assay procedure [28,29]. Minerals were measured with a flame atomic absorption spectrometer at the Analysis of Soils, Plants and Water Service in the Institute of Agricultural Sciences, Madrid (Spain). The caloric value of the loaves was calculated using the Atwater coefficient based on the caloric coefficient corresponding to the protein, carbohydrate and lipid contents.

### 2.4. Determination of Myo-Inositol Phosphates

Ins*P*_6_ (phytic acid or phytates) present in the raw materials and the remaining Ins*P*_6_ and lower *myo*-inositol phosphates generated during the breadmaking process (Ins*P*_5_, Ins*P*_4_, and Ins*P*_3_) were purified by ion-exchange chromatography and measured by the HPLC (High Performance Liquid Chromatography) method in reverse phase described by Türk et al. [30], as modified by Sanz-Penella et al. [31]. The *myo*-inositol phosphates were identified by comparison with standards of phytic acid di-potassium salt (Sigma-Aldrich, St. Louis, MO, USA). Samples were analyzed in triplicate.

### 2.5. Amino Acid Composition

The amino acid composition of the samples tested was analyzed by reverse phase liquid chromatography after acid hydrolysis according to AOAC (Association of Official Analytical Chemists) 994.12 and derivatized with diethyl ethoxymethylenemalonate to obtain the amino acids compound *N*-(2,2-bis(ethoxycarbonyl) vinyl) [32]. Tryptophan was determined by basic hydrolysis and neutralization and analysis by reverse-phase HLPC with spectrophotometric determination, using an isocratic elution system consisting of sodium acetate and sodium azide/acetonitrile, according to Yust et al. [33].

### 2.6. Amino Acid Score

The amino acid score (AAS) was obtained by dividing the amino acid content of the raw materials or bread (mg/g protein) by the scoring pattern for children (1–2-years-old) given by FAO/WHO/UNU [13] and EFSA [12], according to Equation (1):

(1) $$\mathrm{AAS}=\frac{\mathrm{mg}\;\mathrm{of}\;\mathrm{amino}\;\mathrm{acid}\;\mathrm{in}\;1\;g\;\mathrm{test}\;\mathrm{protein}}{\mathrm{mg}\;\mathrm{of}\;\mathrm{amino}\;\mathrm{acid}\;\mathrm{in}\;\mathrm{requirement}\;\mathrm{pattern}}$$

### 2.7. Fatty Acid Profile

The samples were transesterified to convert triglycerides into fatty acid methyl esters (FAMEs), following the methodology previously described by Garces and Mancha [34]. The fatty acid composition and quantification were analyzed by GC (Gas chromatography) (7890A; Agilent, Santa Clara, CA, USA) fitted with a capillary column (30 m length; 0.32 mm internal diameter; 0.20 µm film thickness) of fused silica (Supelco, Bellafonte, PA, USA) and a flame ionization detector according to International Union of Pure and Applied Chemistry (IUPAC) method 2.302 [35]. Measurements were carried out in triplicate.

### 2.8. Estimation of In Vitro Glycemic Index

To evaluate the in vitro rate of starch hydrolysis, the method described by Goni et al. [36] was employed, with slight modifications according to Sanz-Penella et al. [37]. The hydrolysis index (HI) of the samples was calculated from the area under the curve (AUC) from 0 to 120 min as a percentage of the corresponding reference area (wheat bread; HI = AUCsample/AUCwheat bread × 100). The glycemic index (GI) was calculated using the equation GI = 0.549 × HI + 39.71. The measurements were carried out in triplicate. The predicted glycemic load (pGL) for a 100 g bread portion was calculated from the glucose-related GI, with pGL = glycemic index × total carbohydrates/100, taking into account the total carbohydrates of each sample [38].

### 2.9. Statistical Analysis

Multiple sample comparison of the means (ANOVA) and Fisher’s least significant differences (LSD) were applied to establish significant statistical differences between treatments. All statistical analyses were carried out with the Statgraphics Centurion XV.II software (Virginia, VA, USA), and the significance level was established at *p* < 0.05.

## 3. Results and Discussion

The protein and ash contents in CSDF and CLFF were higher than in chia seeds or whole chia flour, while the lipid contents were lower, as was expected after the defatting process. A similar trend was found by Capitani et al. [3], where the nutrients were concentrated after the extraction of chia oil. The bread with chia seeds and chia-by products showed a significant (*p* < 0.05) increase in the levels of ash, total dietary fiber (TDF), lipids, and proteins, and a decrease (*p* < 0.05) in the starch content in comparison with the control bread (Table 1, Table 2, Table 3, Table 4 and Table 5).

### 3.1. Evaluation of Quality Proteins

The levels of proteins in the raw materials were in the following descending order, CLFF > CSDF > CWF > CWS > W, with the chia flours after oil extraction (CLFF and CSDF) showing significantly higher protein contents than the wheat flour and chia, as was expected (Table 1). On the other hand, the protein content in CWS (20.2 ± 0.2 g/100 g d.m.) was higher than in other oilseeds, such as sunflower (19.33 g/100 g) and sesame seed (17.73 g/100 g), and even than chia seed in other studies (16.54 g/100 g) [39]. This variation can be attributed to various factors, such as growing region, stage of plant development, genotype, temperature, light, and soil [40]. As mentioned above, the protein contents in the defatted chia flours CSDF (22.5 ± 0.5 g/100 g) and CLFF (23.5 ± 0.1 g/100 g d.m.) were significantly higher. These values could vary, depending on the defatting process or type of oilseed, for example, but they were higher than in sesame by-products after seed defatting and dehulling (10.23 ± 0.32 g/100 g d.m.), and lower than in defatted flax by-product (which ranged from 35% to 40%) [41].

The nutritional contribution of food proteins to the maintenance of consumer health depends on their biological quality, given by the presence of all the essential amino acids (EAAs) [13,42]. The chia seed and chia flours had up to two times more EAAs than wheat flour, with the highest values corresponding to defatted and semi-defatted chia flour (Table 1). In the current investigation, the total amount of essential amino acids in chia seeds was 38%, similar to the results found in the literature [11,43,44]. There were notable increases in the amounts of tyrosine (Tyr), histidine (His), methionine (Met), tryptophan (Trp), lysine (Lys), and cysteine (Cys) after the defatting process, as can be seen in the CSDF and CLFF samples in comparison with the chia seeds and whole chia flour (Table 1). Furthermore, with regard to the stability of certain amino acids, the defatting process could be advantageous because of the high susceptibility of lipids to oxidation. The generation of lipid-free radicals can induce the release of protein-free radicals, which form protein–protein or lipid–protein complexes. Moreover, lipid oxidation products, such as peroxides and hydroperoxides, could damage amino acid residues [45].

**Figure 1:**
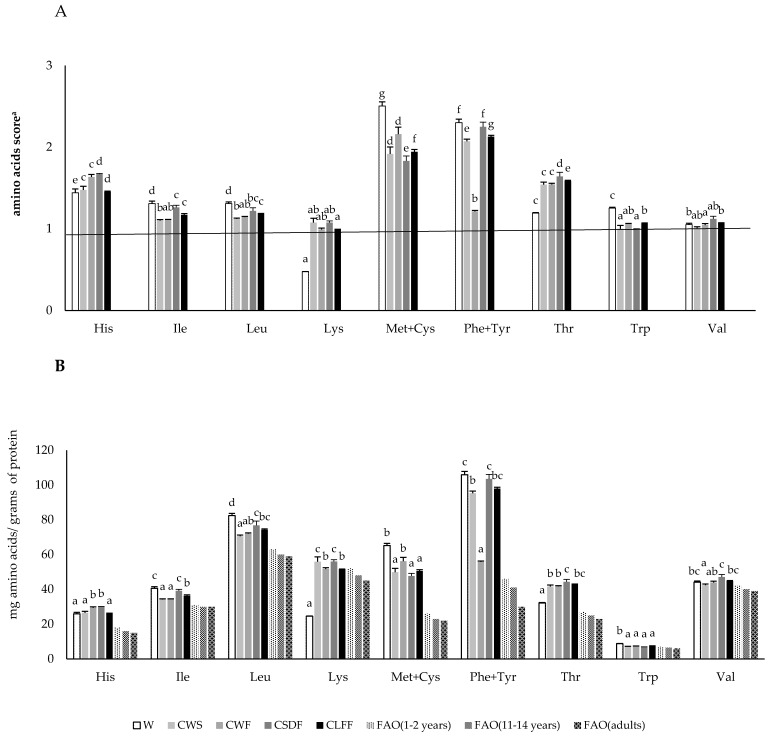
Evaluation of essential amino acids in chia ingredients. (**A**) Amino acids score (mg/g protein): essential amino acid pattern requirement for children as high-quality protein. (**B**) Composition of amino acids (mg/g protein d.m.): based on FAO/WHO/UNU (Food and Agriculture Organization/World Health Organization/United Nations University) standard (2007), 1–2-year-old reference pattern (mg/g protein): His-18, histidine; Lys-52, lysine; Ile-31, isoleucine; Leu-63, leucine; Met+Cys-26, methionine + cysteine; Phe+Tyr-46, phenylalanine + tyrosine; Thr-27, threonine; Trp-7, Tryptophan; Val-42, Valine. Wheat flour (W); CWS, whole chia seed; CWF, whole chia flour; CSDF, semi-defatted chia flour; and CLFF, low-fat chia flour. Values are expressed as mean ± standard deviation (*n* = 3). Bars followed by the same letter are not significantly different at 95% confidence level.

Given that protein quality is to directly associated with the essential amino acid profile, it is important to note that the scores for histidine, isoleucine, leucine, lysine, methionine+cysteine, phenylalanine+tyrosine, threonine, tryptophan, and valine in the chia protein ingredients were higher than in the protein reference pattern for children (1–2-years-old) and adults [12,13]. In the case of lysine, which is the limiting amino acid in cereals, the score in the chia ingredients was around 1 (Figure 1A). On the other hand, the non-essential amino acids in the chia ingredients had abundant amounts of glutamic acid+glutamine (37.8–46.7 mg/g), arginine (19.6–23.4 mg/g), and aspartic acid+asparagines (17.9–21.8 mg/g), corresponding to 60% of the amount of non-essential amino acids, which was similar to the percentage observed in defatted chia flour [44] and chia seed [46].

In other studies, chia seeds and defatted flour contained limiting amino acids such as threonine, lysine, and leucine [47,48], but in the current study limiting amino acids did not appear. This discrepancy could be due to the different varieties, soils, and climatic conditions of the crop, as was reported by Ayerza [49]. However, the chia proteins in this study contained all the essential amino acids in quantities corresponding to human requirements according to the scoring patterns for the 1–2-year-old, 11–14-year-old, and adult age groups [12,13]. The wheat protein, as is also the case with whole wheat flour [50], showed a deficient protein quality for all age groups in comparison with the chia proteins (Figure 1B), mainly because of the low lysine content. Besides, the purpose of food made with nutritional ingredients is to contribute to the recommended dietary allowance (RDA) of each nutrient, taking into account the age group and body weight when setting the daily consumption [12,13]. In the case of proteins/amino acids, intake of 15 g of chia seeds or chia whole flour (3 g of protein) for an adult weighing 70 kg would provide 7% of the adult RDA of Ile, Try, Val, and Lys, 17% of the adult RDA of Met+Cys, and 21% of the adult RDA of Phe+Tyr. Taking into account the recommendation of EFSA (see Introduction), intake of 12 g of the defatted chia flours (CSDF/CLFF; 2.6 g of protein) would provide 6% of the adult RDA of Lys and Val, 13% of the adult RDA of Met+Cys, and 19% of the adult RDA of Phe+Tyr in the same individual. Consequently, chia ingredient intake could provide a high percentage of the adult RDA of sulfur and aromatic amino acids in the diet.

The bread formulations with chia ingredients showed significantly higher protein contents than the wheat bread, particularly in formulations with 10% substitution (Table 1).

Intake of 100 g of bread with 5% of chia by a 70 kg adult who performs normal activity could cover 19% of the PRI (popular reference intake; EFSA, 2017) or RDA (recommended dietary allowance; FAO/WHO/UNU, 2007) of protein, similar to the contribution of the control bread (18%). The contribution of protein to the PRI would depend on the ingredient and level in the formula, all of which provided a better contribution than the control sample (Table 1).

Taking in account the amino acid composition, although the chia seed and flours had a higher amount of essential amino acids than the wheat flour, the bread formulations with 5% and 10% replacement showed only a slight increase compared to the control bread (Table 1).

The presence of abundant hydrophobic interactions of chia proteins, which could delay their denaturalization in a thermal process [47], could explain why no lysine losses were observed in the bread formulations (Table 1). However, in bread enriched with legumes there was a reduction of nutritional quality in terms of essential amino acids after the breadmaking process, especially with regard to lysine, which reacts with reducing carbohydrates to form amino acid–sugar compounds, which could not make up for the deficiency of lysine in the control bread [51]. There were slight increases in the amounts of methionine and histidine in comparison with the control bread, although they decreased or remained constant when the proportion of chia increased (Table 1). This behaviour was also observed by Oğur [52] when she evaluated the changes in the amounts of amino acids in bread with partial replacement of flour by washed fish mince. The reduction of some amino acids may have been due to reactions with other bread components, as in Maillard reactions. Amino acids are consumed during fermentation, and then their concentration increases at the end of this stage as a result of yeast activity, which adapts to the nutritional conditions of the medium. Moreover, there are factors, such as fermentation time and dough pH, that can affect the amounts of amino acids during fermentation [52,53]. With regard to the amounts of non-essential amino acids, there were significant increases in the amounts of glutamic acid + glutamine, glycine, arginine, and alanine in the breads with 5% and 10% replacement, particularly in the formulations with chia flour from which oil had been extracted (CSDF and CLFF). There was a decrease in proline in all the formulations with chia ingredients, as was expected because of the lower amount of this amino acid in the raw materials in comparison with wheat flour, in agreement with results reported by Diana et al. [54] and Turfani et al. [51].

### 3.2. Evaluation of Fatty Acid Profile

The chia seeds and flour had higher lipid concentrations than the wheat flour, as was expected (Table 2). In addition, chia oil had a higher (*p* < 0.05) amount of saturated fatty acids (SFAs) than wheat oil, in which they were mainly palmitic and stearic acids (Table 2). Of course, the defatting process affected the amount of fatty acids and may have affected the fatty acid profile of the CSDF and CLFF chia ingredients (Table 2). In the semi-defatted chia flour the amount of PUFAs was still higher than the amount of SFAs, consisting mainly of linoleic and α-linolenic acids, just as in the chia seeds and whole flour. Moreover, the defatting process increased the PUFA:SFA ratio from 3.1:1/3.6:1 (chia flour/chia seeds) to 4.3:1 (CSDF) and 5.4:1 (CSFF). The efficiency of chia oil extraction and its fatty acid profile depend on the supercritical carbon dioxide extraction conditions such as pressure, temperature, and extraction time [55]. It was observed that extraction time had a significant effect on the percentages of linoleic acid (12.7 and 14.3 g/100 g of chia oil) and linolenic acid (32.8 and 34.7 g/100 g of chia oil) and on the PUFA:SFA ratios (4.3 and 5.4) and the ω-3/ω-6 ratios (2.6 and 2.4) in the CSDF and CLFF samples. As the unsaturated fatty acids (PUFAs) were much more concentrated in the chia seeds and flours than in the wheat flour, better ω-3/ω-6 ratios were observed in the chia seeds and chia flours (between 3.0 and 2.4) than those recommended by WHO/FAO [6] or EFSA [7] (1:5 and 1:8, respectively). A prolonged diet with a low ω-3/ω-6 ratio could lead to the development of chronic diseases such as cardiovascular disease, cancer, and inflammatory and autoimmune diseases [56]. Accordingly, the inclusion of chia ingredients in food formulations could be a good strategy to promote the intake of healthy lipids, which could help to reduce the risk of developing diseases, have a beneficial effect on brain function, and help to avoid cardiovascular disease, arthritis and some types of cancer [57]. In addition, there are studies on experimental animals and humans in which intake of chia seeds reduced the plasma triglycerides level, owing to their high α-linolenic content [58,59]. However, the stability of these unsaturated fatty acids is sometimes compromised, depending on the process used, as explained below.

**Table 1:** Amino acid composition of raw materials used in this study, mg/g of bread in dry matter ^a^.

Amino Acid	Raw Materials					Bread Formula								
	Wheat (W)	Chia Whole Seeds (CWS)	Chia Whole Flour (CWF)	Chia Semi-Defatted Flour (CSDF)	Chia Low-Fat Flour (CLFF)	Control (WB)	Chia Seeds (CWSB)		Chia Whole Flour (CWFB)		Chia Semi-Defatted Flour (CSDFB)		Chia Low-Fat Flour (CLFFB)	
							5%	10%	5%	10%	5%	10%	5%	10%
Proteins, % d.m.	10.1 ± 0.1 a	20.2 ± 0.2 b	20.0 ± 0.1 b	22.5 ± 0.5 c	23.5 ± 0.1 d	16.1 ± 0.1 a	16.87 ± 0.5 b	22.4 ± 0.1 c	17.09 ± 0.4b	24.0 ± 0.1 d	16.93 ± 0.1 b	24.3 ± 0.2 e	17.24 ± 0.5 b	25.2 ± 0.1 f
**Essential Amino Acids (EAA)**														
Histidine	2.6 ± 0.1 a	5.2 ± 0.2 b	5.8 ± 0.2 c	6.7 ± 0.1 d	6.1 ± 0.1 c	2.4 ± 0.0 a	2.9 ± 0.2 bc	2.9 ± 0.0 bc	2.9 ± 0.2 bc	2.7 ± 0.0 b	3.1 ± 0.0 c	3.0 ± 0.1 bc	3.1 ± 0.1 c	2.9 ± 0.1 bc
Threonine	3.2 ± 0.0 a	8.3 ± 0.3 b	8.3 ± 0.1 b	9.7 ± 0.4 c	10.0 ± 0.1 c	3.6 ± 0.0 a	3.9 ± 0.1 b	4.1 ± 0.0 de	3.8 ± 0.1 b	4.2 ± 0.0ef	4.0 ± 0.1 c	4.3 ± 0.0 f	4.1 ± 0.0 cd	4.2 ± 0.0 ed
Tyrosine	4.9 ± 0.1 a	8.2 ± 0.3 b	8.3 ± 0.2 b	9.5 ± 0.5 c	9.8 ± 0.1 c	4.3 ± 0.0 a	4.9 ± 0.1 b	5.0 ± 0.0 b	5.0 ± 0.4 b	4.9 ± 0.1b	5.1 ± 0.1 b	5.7 ± 0.1 c	5.1 ± 0.2 b	5.2 ± 0.3 b
Valine	4.4 ± 0.1 a	8.5 ± 0.2 b	8.7 ± 0.2 b	10.4 ± 0.5 c	10.5 ± 0.1 c	4.7 ± 0.1 a	5.2 ± 0.1 bcd	4.9 ± 0.1 ab	5.2 ± 0.0 bcd	4.9 ± 0.3 ab	5.5 ± 0.0 cde	5.6 ± 0.1 de	5.9 ± 0.0 e	5.1 ± 0.5 abc
Methionine	1.9 ± 0.1 a	3.2 ± 0.5 b	4.6 ± 0.3 c	3.4 ± 0.2 b	4.1 ± 0.2 bc	2.1 ± 0.1 ab	2.5 ± 0.1 abc	2.6 ± 0.4 abc	2.9 ± 0.1 c	2.0 ± 0.4 a	2.8 ± 0.2 bc	2.8 ± 0.1 c	3.1 ± 0.4 c	2.1 ± 0.3 b
Phenylalanine	5.7 ± 0.1 a	10.9 ± 0.0 b	11.1 ± 0.2 b	13.4 ± 0.3 c	13.1 ± 0.1 c	5.8 ± 0.0 a	6.2 ± 0.1 bc	6.4 ± 0.1 cd	6.2 ± 0.1 b	6.7 ± 0.2 ef	6.5 ± 0.0 de	6.7 ± 0.1 f	6.6 ± 0.0 def	6.6 ± 0.1 def
Tryptophan	0.9 ± 0.0 a	1.3 ± 0.1 b	1.5 ± 0.0 c	1.6 ± 0.0 d	1.8 ± 0.0 d	0.7 ± 0.0 b	0.4 ± 0.0 a	1.0 ± 0.0 c	0.4 ± 0.1 a	1.0 ± 0.0 c	0.4 ± 0.0 a	1.0 ± 0.1 c	0.4 ± 0.0 a	1.0 ± 0.1 c
Isoleucine	4.0 ± 0.1 a	6.8 ± 0.1 b	6.8 ± 0.1 b	8.7 ± 0.3 c	8.4 ± 0.2 c	4.1 ± 0.1 a	4.4 ± 0.1 abc	4.1 ± 0.1 a	4.4 ± 0.1 abc	4.0 ± 0.3 a	4.7 ± 0.0 cd	4.6 ± 0.0 bc	5.1 ± 0.0 d	4.3 ± 0.5 ab
Leucine	8.2 ± 0.2 a	14.2 ± 0.2 b	14.3 ± 0.2 b	16.9 ± 0.8 c	17.4 ± 0.2 c	8.4 ± 0.0 a	8.9 ± 0.2 bc	8.9 ± 0.0 bc	8.7 ± 0.1 b	9.1 ± 0.1 cd	9.2 ± 0.0 cd	9.6 ± 0.2 e	9.5 ± 0.0 e	9.3 ± 0.2 de
Lysine	2.5 ± 0.0 a	10.9 ± 0.8 bc	10.2 ± 0.2 b	12.4 ± 0.4 d	12.1 ± 0.1 cd	2.0 ± 0.1 a	3.4 ± 0.0 b	3.6 ± 0.0 c	3.4 ± 0.1 b	3.8 ± 0.1 cd	3.6 ± 0.0 c	3.9 ± 0.1 d	3.8 ± 0.1 cd	3.9 ± 0.1 d
**Total EAAs**	38	78	80	93	93	39	43	44	43	43	45	47	47	45
**Non-Essential Amino Acid (NEAA)**														
Aspartic acid + asparagine	4.6 ± 0.0 a	17.9 ± 0.6 b	17.7 ± 0.1 b	20.0 ± 0.7 c	21.8 ± 0.3 d	5.2 ± 0.1 a	6.1 ± 0.2 bc	6.9 ± 0.1 ef	5.8 ± 0.1 b	7.1 ± 0.1 fg	6.3 ± 0.2 cd	7.5 ± 0.2 h	6.7 ± 0.3 de	7.3 ± 0.1 gh
Glutamic acid + glutamine	38.8 ± 0.5 a	37.8 ± 0.6 a	37.4 ± 0.3 a	43.1 ± 1.0 b	46.7 ± 0.5 c	38.2 ± 0.3 ab	38.9 ± 0.8 bc	38.9 ± 0.5 bc	38.0 ± 0.2 a	39.0 ± 0.2 bcd	39.8 ± 0.2 de	40.1 ± 0.2 e	39.2 ± 0.1 cd	40.2 ± 0.3 e
Serine	6.7 ± 0.1 a	14.5 ± 0.5 b	14.2 ± 0.1 b	16.0 ± 0.6 c	17.6 ± 0.1 d	6.8 ± 0.1 ab	7.1 ± 0.0 ab	7.8 ± 0.1 c	6.8 ± 0.2 a	7.9 ± 0.1 c	7.2 ± 0.1 b	8.3 ± 0.4 d	7.2 ± 0.1 ab	7.9 ± 0.1 c
Glycine	4.4 ± 0.0 a	11.6 ± 0.4 c	10.7 ± 0.2 b	12.1 ± 0.2 c	13.6 ± 0.1 d	4.6 ± 0.0 a	4.9 ± 0.2 bc	5.3 ± 0.1 e	4.7 ± 0.0 ab	5.3 ± 0.0 e	5.0 ± 0.1 cd	5.4 ± 0.0 e	5.1 ± 0.1 d	5.5 ± 0.1 e
Arginine	4.2 ± 0.2 a	19.6 ± 0.7 b	22.0 ± 0.4 c	26.3 ± 0.5 d	23.4 ± 1.0 c	4.7 ± 0.0 a	5.8 ± 0.2 c	6.5 ± 0.0 d	5.5 ± 0.2 bc	6.3 ± 0.2 d	5.8 ± 0.2 c	6.4 ± 0.0 d	5.4 ± 0.1 b	6.4 ± 0.2 d
Alanine	3.9 ± 0.1 a	11.8 ± 0.2 bc	11.1 ± 0.3 b	12.5 ± 0.4 c	14.7 ± 0.4 d	4.0 ± 0.0 a	4.4 ± 0.1 a	5.0 ± 0.1 bc	4.4 ± 0.0 a	5.2 ± 0.1 bc	4.8 ± 0.2 b	5.7 ± 0.4 bc	5.2 ± 0.1 c	5.3 ± 0.2 c
Proline	10.3 ± 0.9 c	5.9 ± 0.4 ab	5.2 ± 0.1 a	7.0 ± 0.1 b	7.7 ± 0.5 b	11.0 ± 0.6 d	6.7 ± 0.4 a	6.3 ± 0.2 a	8.3 ± 0.4 b	7.9 ± 0.4 b	9.9 ± 0.6 c	8.3 ± 0.1 b	8.4 ± 0.0 b	7.7 ± 0.0 b
Cysteine	4.6 ± 0.1 a	6.5 ± 0.2 b	6.3 ± 0.3 b	7.1 ± 0.3 bc	7.7 ± 0.1 c	3.9 ± 0.0 a	4.1 ± 0.1 ab	4.4 ± 0.1 abc	4.3 ± 0.5 abc	4.4 ± 0.2 bc	4.1 ± 0.2 ab	4.7 ± 0.2 c	4.0 ± 0.0 ab	4.7 ± 0.1 c
**Total NEAAs**	77	126	125	144	153	78	78	82	78	83	83	86	81	85

^a^ Values are expressed as mean ± standard deviation (*N* = 3), values followed by the same letter in the same row are not significantly different at 95% confidence level. The statistical analysis of the raw materials was carried out separately from the statistical analysis of the bread samples, d.m. dry matter.

One of the highest values of monounsaturated acid (MUFA) was found in WB, consisting mainly of oleic and elaidic acids. In general, the MUFA content in the bread with wheat flour partially replaced by chia was less than in the control sample, with the exception of the sample CWSB10 (Table 2). It is well known that unsaturated fatty acids are affected by high temperature and the presence of oxygen as a result of the breadmaking process, because the double bonds in their chemical structure are sensitive to oxidation [45]. Consequently, the level of PUFAs in the bread with wheat flour partially replaced by CWF, CSDF, or CLFF was much less than in the bread with wheat flour partially replaced by chia seeds (Table 2). This could be due to protection of lipids by the seed outer cover structure, which remained intact throughout the whole process. However, all the formulations with chia had a significantly higher amount of PUFAs than in the control bread, mainly due to the α-linolenic acid (ALA) concentration (*p* < 0.05). Coelho and Salas-Mellado found that bread made with partial replacement of wheat flour by chia flour had lower SFA amounts and higher PUFA amounts than control bread in more drastic baking conditions, 20 min at 220 °C, than those used in the current investigation [43]. In general, the substitution of wheat flour by chia flour produced a worse ω-3/ω-6 ratio than in the chia seed bread, regardless of the proportion of substitution, and even more so in the case of the defatted chia flours, in which the ratio decreased from 1:1 to 1:8 (Table 2). However, they were better than in the control bread and are near the recommended values [6,7]. Dietary recommendations (adequate intake (AI)) exist for polyunsaturated fatty acids, which are expressed as a percentage of total dietary energy (E%) [24]. The AI for linoleic acid (LA) is 4 E% and for α-linolenic acid (ALA) it is 0.5 E% [24]. The amounts of LA and ALA in the breads with chia made a greater contribution to dietary AI E% than the control bread, and CWSB10 made a higher contribution to the AI E% of LA (~6%) and ALA (~46%) than the other bread formulations, assuming an intake of 100 g of bread.

On the other hand, the caloric values of the breads with 5% replacement of wheat flour by chia ingredients presented a range between 256 and 270 kcal/100 g (CLFFB5 and CSDFB5, respectively), whereas the values of the breads with 10% replacement were slightly lower (232–268 kcal/100 g). CSDFB10 (232 kcal/100 g) and CLFFB10 (237 kcal/100 g) had lower values than the control bread (254 kcal/100 g), and also than the bread with chia, flax and sesame seeds (250 kcal/100 g) reported by USDA (United States Department of Agriculture) [10]. The main component in semi-defatted or low-fat chia flour is mucilage, so the breads made with those flours had the lowest values, as was expected. A similar trend was found by Fernandes and Salas-Mellado [1], who made breads and cakes with vegetable fat replaced by chia mucilage, which decreased the caloric value.

### 3.3. Mineral Contribution to Popular Reference Intake/Recommended Dietary Allowances (PRI/RDAs) and Prediction of Mineral Bioavailability

The ash content in the chia ingredients was higher than in the wheat flour, and the ash contents of the semi-defatted and low-fat chia flours were approximately two times greater than the ash content of the chia flour. Furthermore, the higher levels of minerals found in the raw chia flours in comparison with wheat flour led to higher levels of these nutrients in the bread samples, as was expected (Table 3). The substitution of wheat flour by chia ingredients contributed between two and four times more Ca, Fe, and Zn than in the control bread (data not shown). Accordingly, if mineral absorption inhibitors are absent, the incorporation of chia in bread could increase the percentage contribution of important minerals, assuming an average intake of 100 g of bread per day, according to the PRI (popular reference intake [24]) or the recommended dietary allowances (RDAs)). Ca was the only case in which the PRI contribution of the CLFFB formulation was higher (12–14%) than the contribution of the control bread (9%). The same tendency was observed in the case of Fe, for which the contribution to PRI of bread with chia was higher than that of the control bread, and the formulation with CLFF was the best (Table 3). However, Ca, Fe, and Zn would not be bioavailable in their entirety because of the high presence of phytates, as shown by the Ins*P*_6_/mineral molar ratio, which can predict the effect of phytate on the absorption of minerals [60]. High levels of phytates were found in the chia ingredients (5.4–6.6 µmol/g on dry basis; Table 3). It is widely known that phytates have adverse effects on the bioavailability of di- and trivalent cations because of the formation of insoluble complexes in the intestinal tract of human and monogastric animals. However, there is a decrease in phytate content during the breadmaking process, mainly caused by the activity of phytase, which depends on many factors, such as temperature and pH, among others. Despite this reduction, it is generally not enough to make much improvement in the bioavailability of some minerals [31]. Consequently, the high phytate levels in the bread with 5% or 10% of chia could affect the mineral bioavailability of Zn, Fe, and Ca, as predicted by the phytate/mineral molar ratios [31].

**Table 2:** Fatty acid composition of raw material and bread with 5% and 10% replacement used in this study, g/100 g d.m. ^a^.

Fatty Acid	Raw Materials					Bread Formula								
	Wheat (W)	Seeds (CWS)	Whole Flour (CWF)	Semi-Defatted Flour (CSDF)	Chia Low-Fat Flour (CLFF)	Control (WB)	Chia Seeds (CWSB)		Chia Whole Flour (CWFB)		Chia Semi-Defatted Flour (CSDFB)		Chia Low-Fat Flour (CLFFB)	
							5%	10%	5%	10%	5%	10%	5%	10%
**Lipids, % d.m.**	2.09 ± 0.3 a	39.3 ± 1.3 d	41.5 ± 2.3 d	20.6 ± 0.7 c	13.5 ± 0.2 b	1.78 ± 0.1 bc	2.88 ± 0.1 de	3.5 ± 0.0 e	1.44 ± 0.2 abc	1.88 ± 0.01 c	1.39 ± 0.15 ab	1.44 ± 0.3 abc	1.14 ± 0.02 a	1.68 ± 0.1 bc
**∑ SFA**	0.53	3.36	4.33	2.16	1.23	0.54	0.58	0.61	0.49	0.60	0.54	0.52	0.44	0.50
Palmitic acid (C16:0)	0.48 ± 0.00 a	2.11 ± 0.04 d	2.65 ± 0.12 e	1.34 ± 0.05 c	0.83 ± 0.05 b	0.38 ± 0.00 abc	0.43 ± 0.01 c	0.43 ± 0.02 bc	0.39 ± 0.02 abc	0.43 ± 0.03 bc	0.40 ± 0.02 bc	0.39 ± 0.02 abc	0.35 ± 0.02 a	0.38 ± 0.04 ab
Stearic acid (C18:0)	0.02 ± 0.02 a	1.08 ± 0.00 d	1.46 ± 0.10 e	0.72 ± 0.00 c	0.40 ± 0.02 b	0.14 ± 0.01 efg	0.13 ± 0.00 def	0.15 ± 0.02 g	0.09 ± 0.00 ab	0.15 ± 0.01 fg	0.12 ± 0.01 cde	0.11 ± 0.01 cd	0.07 ± 0.00 a	0.10 ± 0.00 bc
Arachidic acid (C20:0)	0.02 ± 0.00 a	0.12 ± 0.00 c	0.15 ± 0.01 d	0.07 ± 0.00 b	n.d.	0.02 ± 0.00 c	0.01 ± 0.00 bc	0.02 ± 0.00 c	0.01 ± 0.00 abc	0.01 ± 0.00 bc	0.01 ± 0.00 abc	0.01 ± 0.00 abc	0.01 ± 0.00 a	0.01 ± 0.00 ab
Behenic acid (C22:0)	0.01 ± 0.00 a	0.05 ± 0.01 c	0.07 ± 0.07 c	0.03 ± 0.00 b	n.d.	n.d.	0.01 ± 0.00 a	0.01 ± 0.00 a	n.d.	0.01 ± 0.00 a	0.01 ± 0.00 a	0.01 ± 0.00 a	0.01 ± 0.00 a	0.01 ± 0.00 a
**∑ MUFA**	0.77	4.63	5.93	2.12	0.72	0.78	0.47	0.80	0.35	0.54	0.43	0.44	0.25	0.51
Elaidic acid (C18:1n9t)	0.15 ± 0.06 b	0.81 ± 0.02 c	0.97 ± 0.09 d	0.27 ± 0.02 b	n.d.	0.10 ± 0.01 cd	0.10 ± 0.01 cd	0.17 ± 0.00 f	0.06 ± 0.00 b	0.12 ± 0.01 e	0.09 ± 0.01 c	0.11 ± 0.01 d	0.03 ± 0.00 a	0.10 ± 0.01 cd
dOleic acid (C18:1n9c)	0.62 ± 0.13 a	3.82 ± 0.08 d	4.96 ± 0.43 e	1.85 ± 0.13 c	0.72 ± 0.05 b	0.68 ± 0.03 e	0.37 ± 0.03 cd	0.63 ± 0.02 e	0.29 ± 0.01 b	0.42 ± 0.04 d	0.34 ± 0.03 bc	0.33 ± 0.00 bc	0.22 ± 0.00 a	0.41 ± 0.03 d
**∑ PUFA**	0.79	12.1	13.3	9.37	6.61	0.36	0.7	1.27	0.6	0.44	0.39	0.48	0.45	0.61
Linoleic acid (C18:2n6c)	0.75 ± 0.01 a	3.02 ± 0.07 c	3.81 ± 0.33 d	2.61 ± 0.18 c	1.93 ± 0.12 b	0.34 ± 0.02 a	0.36 ± 0.01 ab	0.65 ± 0.03 c	0.43 ± 0.12 ab	0.31 ± 0.09 a	0.32 ± 0.00 a	0.37 ± 0.01 ab	0.40 ± 0.08 ab	0.54 ± 0.06 bc
α-Linolenic acid (C18:3n3)	0.04 ± 0.00 a	9.09 ± 0.20 d	9.52 ± 0.83 d	6.76 ± 0.46 c	4.68 ± 0.29 b	0.02 ± 0.00 a	0.34 ± 0.00 f	0.62 ± 0.02 g	0.17 ± 0.00 e	0.13 ± 0.03 de	0.07 ± 0.01 bc	0.11 ± 0.01 cd	0.05 ± 0.00 ab	0.07 ± 0.01 bc
PUFA:SFA Ratio	1.3:1	3.6:1	3.1:1	4.3:1	5.4:1	0.66	1.21	2.1	1.22	0.7	0.7	0.92	1.0	1.34
ω-3/ω-6 Ratio Recommended Ratio 1:5 ^1^; 1:8 ^2^	1:19	3.0:1	2.5:1	2.6:1	2.4:1	1:17	1:1	1:1	1:3	1:2	1:5	1:3	1:8	1:8

^a^ Values are expressed as mean ± standard deviation (*N* = 3), values followed by the same letter in the same row are not significantly different at 95% confidence level. The statistical analysis of the raw materials was carried out separately from the statistical analysis of the bread samples, d.m.: dry matter, n.d.: not detected; codes: SFA, saturated fatty acids; MUFA, monounsaturated fatty acids; PUFA, polyunsaturated fatty acids; **^1^** WHO/FAO (World Health Organization/Food and Agriculture Organization), 2010; **^2^** EFSA (European Food Safety Authority), 2010.

**Table 3:** Effect of bread formulation on mineral dietary reference intake contribution and mineral availability prediction.

Parameter *	Units			Wheat Flour (W)	Chia Ingredients							
					Seeds (CWS)		Whole Flour (CWF)		Semi-Defatted (CSDF)		Low-Fat (CLFF)	
Ash ^a^	g 100 g^−1^			0.6 ± 0.0 a	2.0 ± 0.4 b		1.9 ± 0.5 b		3.5 ± 0.0 c		4.9 ± 0.1 d	
Ca ^a^	mg 100 g dm^−1^			106 ± 4 a	524 ± 4 b		659 ± 3 c		860 ± 10 e		805 ± 14 d	
Fe ^a^	mg 100 g dm^−1^			1.5 ± 0.1 a	7.3 ± 0.6 b		10.3 ± 0.2 d		8.0 ± 0.2 bc		8.3 ± 0.0 c	
Zn ^a^	mg 100 g dm^−1^			1.8 ± 0.0 a	8.7 ± 1.1 c		7.0 ± 0.1 b		7.7 ± 0.2 bc		8.1 ± 0.1 bc	
Ins*P*_6_	µmol g dm^−1^			n.d.	5.4 ± 1.2 b		6.6 ± 1.3 c		5.1 ± 1.0 a		6.6 ± 0.7 c	
				**Control Bread** **(WB)**	**Bread with Chia Ingredients**							
		**PRI/RDA, mg day^−1^** **or** **Ins*P*_6_/Mineral, mol mol^−1^**			**Seeds (CWSB)**		**Whole Flour (CWFB)**		**Semi-Defatted (CSDFB)**		**Low-Fat (CLFFB)**	
					**5%**	**10%**	**5%**	**10%**	**5%**	**10%**	**5%**	**10%**
Ash^a^	g 100g^−1^			2.0 ± 0.1 a	2.5 ± 0.1 b	2.7 ± 0.2 b	2.4 ± 0.1 b	2.7 ± 0.2 b	2.5 ± 0.1 b	3.2 ± 0.0 c	2.4 ± 0.2 b	3.7 ± 0.0 d
Ca Contribution	%	FAO ^b^ EFSA ^b^	1,000 950	9 9	12 12	12 13	7 7	12 13	9 10	11 11	12 13	14 15
Fe Contribution	%	FAO ^b^ EFSA ^b^	14/29 11/16	10/5 12/9	11/5 14/10	13/6 17/11	12/6 16/11	14/7 18/12	13/6 17/11	14/7 18/12	14/7 18/12	15/7 19/13
Zn Contribution	%	FAO ^b^_High bioavailability_ FAO ^b^_Moderate bioavailability_ FAO ^b^_Low bioavailability_ EFSA ^b^_300_ EFSA ^b^_600_ EFSA ^b^_900_ EFSA ^b^_1200_	4.2/3 7/4.9 14/9.8 9.4/7.5 11.7/9.3 14/11 16.3/12.7	40/56 23/19 12/17 18/22 14/18 12/15 10/13	43/61 25/20 13/19 19/24 16/20 13/17 11/14	53/75 30/25 16/23 24/30 19/24 16/20 14/18	49/69 28/23 15/21 22/27 18/22 15/19 13/16	50/70 28/23 15/21 22/28 18/22 15/19 13/16	48/67 27/22 14/21 21/27 17/22 14/18 12/16	50/70 28/23 15/21 22/28 18/22 15/19 13/16	50/69 28/23 15/21 22/28 18/22 15/19 13/16	54/75 30/25 16/23 24/30 19/24 16/21 14/18
Ins*P*_6_	µmol g dm^−1^			n.d	1.2 ± 0.3 a	4.1 ± 0.8 bc	0.8 ± 0.3 a	3.6 ± 0.3 b	0.9 ± 0.2 a	3.6 ± 0.4 b	0.9 ± 0.1 a	4.8 ± 0.6 c
Ins*P*_5_	µmol g dm^−1^			n.d	0.18 ± 0.02 a	1.1 ± 0.1 c	0.10 ± 0.05 a	0.76 ± 0.06 b	0.12 ± 0.03 a	0.76 ± 0.13 b	0.1 ± 0.00 a	0.83 ± 0.08 b
Ins*P*_4_	µmol g dm^−1^			n.d	0.04 ± 0.01 a	0.38 ± 0.04 e	0.06 ± 0.09 a	0.37 ± 0.02 cd	0.04 ± 0.04 a	0.33 ± 0.04 be	0.05 ± 0.05a	0.30 ± 0.03 b
Ins*P*_3_	µmol g dm^−1^			n.d	0.05 ± 0.01 a	0.20 ± 0.03 cd	0.27 ± 0.09 cd	0.53 ± 0.02 e	0.16 ± 0.04 b	0.30 ± 0.03 d	0.22 ± 0.05 bc	0.31 ± 0.04 d
Ins*P*_6/_Ca ^c^	mol mol^−1^		<0.24	0	0.03	0.11	0.03	0.09	0.03	0.10	0.02	0.09
Ins*P*_6/_Fe ^c^	mol mol^−1^		<1	0	3.4	10.0	1.8	7.4	2.0	7.1	2.0	9.1
Ins*P*_6_/Zn ^c^	mol mol^−1^		<5	0	3.4	9.54	1.8	8.13	2.0	7.89	2.0	9.87

* Values are expressed as mean ± standard deviation (*N* = 3). ^a^ Values followed by the same letter in the same row are not significantly different at 95% confidence level; d.m.: dry matter; n.d. not detected; ^b^ FAO (Food and Agriculture Organization)/RDAs (recommended dietary allowances); EFSA (European Food Safety Authority)/PRIs (popular reference intakes) contribution (%) for a daily average intake of 100 g of bread if mineral absorption inhibitors are absent. PRIs/RDAs in mg per day for males (M)/females (F) ≥18. The FAO considers three levels of bioavailability of zinc, depending on the phytate (Ins*P*_6_) content in the diet: high, FAO_high_ (Ins*P*_6_/mineral < 5); moderate, FAO_moderate_ (Ins*P*_6_/mineral 5–15); and low bioavailability, FAO_low_ (Ins*P*_6_/mineral > 15) [61]. EFSA contemplates four levels of phytate intake per day (300, EFSA_300_; 600, EFSA_600_; 900, EFSA_900_; and 1200 mg per day, EFSA_1200_) [24]; ^c^ Threshold ratios (Ins*P*_6_/mineral) for mineral availability inhibition [60]; Ins*P*_6_, *myo*-inositol hexakisphosphate; minerals Ca, Fe, or Zn.

All the calcium present in the chia breads would be bioavailable, because the Ins*P_6_*:Ca molar ratio was lower than 0.24, which means that its bioavailability would not be compromised. However, for the molar ratio found for Ins*P*_6_, Fe was higher than 1.0 (1.8–10), which indicates that these breads would not be good sources of iron, because it would not be bioavailable. With regard to the bioavailability of Zn, FAO reported it in terms of three categories (high, moderate, and low) [61], whereas EFSA classified it according to the amount of phytate present in the diet (300, 600, 900, or 1200 mg/day) [24]. For the breads with 5% of chia had Ins*P_6_*, Zn molar ratios that were <5, and they could provide at least 50% of the RDAs (FAO high bioavailability). With respect to the chia bread with 10% replacement had Ins*P*_6_, Zn molar ratios between 5 and 15, which corresponds to FAO moderate bioavailability [61]. It is important to note that the predicted bioavailability of zinc in all the breads formulated with chia ingredients was high or moderate because the Ins*P_6_*:Zn molar ratio was less than 15 [61] (Table 3).

### 3.4. Adequate Intake of Total Dietary Fiber in Breads

The amount of total dietary fiber in the breads with 5% or 10% of chia seed or chia flour varied between 6.1% and 8.7%, which was higher than in the control bread (4.1%; Table 4). These results were even higher than those of other formulations of bread with chia (up to 11% of substitution), which had 5.7% of total dietary fiber [43]. The differences could be due to the great differences in the composition of chia seeds, depending on their origin. In the European Union, the current regulations concerning the composition of chia seeds marketed in Europe state that they should have no less than 18% of crude fiber, defined as the part of fiber made mainly of indigestible cellulose, pentosans, and lignin [62]. The amount of dietary fiber in the chia seeds used in the current investigation varied between 30.9% and 36.2%, and was even higher after lipid extraction [16].

Intake of dietary fiber produces physiological activity that is most effective when the soluble/insoluble ratio is 1:2 [63]. The formulations with 10% of chia had ratios close to this value (Table 4). These bakery products could be included in the diet, especially in the diet of people who do not achieve adequate intake of total dietary fiber, and they could have healthy effects such as reducing cholesterol, preventing constipation, and lowering the risk of developing diabetes or cardiovascular disease [64,65]. Most of the fiber in chia seeds is soluble fiber, owing to the high proportion of mucilage, which can absorb up to 35.2 times its weight in water. This water holding capacity increases the viscosity of foods and also of the alimentary bolus, which could delay gastric emptying and thus reduce the accessibility of nutrients such as glucose [14] (Table 4).

From a nutritional point of view, assuming an intake of 100 g of bread per day, the breads with 10% of chia would provide between 33% and 34% of the AI of total dietary fiber for adults, which is 25 g/day [24,66].

### 3.5. Evaluation of Glycemic Index of Bread

The glycemic index could be affected by different factors such as food texture, source of starch, degree of starch gelatinization, and food processing, and by interaction with other ingredients. The control bread showed a high percentage of starch hydrolysis, ~56.9% (at 90 min in the in vitro test), in comparison with the 5% and 10% chia breads (between 5 and 9 units and between 9 and 15 units, respectively). The glycemic index and the glycemic load were lower in the loaves with 10% replacement than in those with 5%, and the latter had lower values than that of the control bread at 90 min in the in vitro test (Table 5). This behaviour corresponds to the lower amount of starch and higher amount of fiber in the bread with 10% substitution than in the bread with 5% substitution or in the control formula.

**Table 4:** Dietary fiber content and contribution to adequate intake in bread formulated with chia ingredients.

Parameter ^a^	Units	Control (WB)	Bread with Chia Ingredients							
			Seeds (CWSB)		Whole Flour (CWFB)		Semi-Defatted (CSDFB)		Low-Fat (CLFFB)	
			5%	10%	5%	10%	5%	10%	5%	10%
Total Dietary Fiber ^a^	g/100g d.m.	4.1 ± 0.1 a	6.1 ± 0.6 b	8.7 ± 0.2 c	6.3 ± 0.2 b	8.5 ± 0.5 c	7.0 ± 0.9 b	8.2 ± 0.1 c	7.1 ± 0.6 b	8.5 ± 0.1 c
Soluble Fiber ^a^	g/100g d.m.	1.0 ± 0.0 a	1.7 ± 0.4 ab	2.4 ± 0.4 bc	1.5 ± 0.2 ab	2.8 ± 0.8 c	1.8 ± 0.3 ab	3.2 ± 0.3 c	1.8 ± 0.0 ab	2.9 ± 0.7 c
Insoluble Fiber ^a^	g/100g d.m.	3.1 ± 0.1 a	4.4 ± 0.5 ab	5.0 ± 0.2 c	4.8 ± 0.0 bc	4.7 ± 0.7 bc	5.1 ± 0.6 bc	5.0 ± 0.4 bc	5.4 ± 0.6 bc	5.6 ± 0.8 bc
Soluble/Insoluble Fiber ratio ^b^	g/g	1:3	1:3	1:2	1:3	1:2	1:3	1:2	1:3	1:2
AI^c^ contribution	%	16	24	35	25	34	28	33	28	34

^a^ Values are expressed as mean ± standard deviation (*N* = 3). Values followed by the same letter in the same row are not significantly different at 95% confidence level. d.m., dry matter; ^b^ Ratio of soluble/insoluble fiber, 1:2 [64]. ^c^ AI (Adequate Intake) contribution (%) for a daily average intake of 100 g of bread. AI in g per day for dietary fiber in adult ≥18 is 25 [24].

Increasing the amount of chia in the bread formulation produced a decrease in the total amount of starch, from 79.1% (control bread) to 76.4–74.2% (5% replacement) and 70.7–72.9% (10% replacement), owing to the dilution effect, since chia ingredients are practically devoid of starch. All the formulations with chia ingredients had a lower GI than that of the control sample. This demonstrates the influence of the chia ingredients on glycemic response. This behaviour could be due to the contribution of the amount of chia mucilage in the bread. A similar trend was found in studies on biscuits fortified with soluble fiber, which had a lower glycemic index than the counterpart without soluble fiber [15]. On the other hand, a significant decrease in the GL (Glycemic Load) value was observed when the proportion of chia seeds in the bread increased from 5% to 10%. There was a significant decrease in the GI of all the bread formulations when the proportion of chia increased from 5% to 10%. Although the bread formulations with chia ingredients had an in vitro GI greater than 70 (high-glycemic food), further studies are needed to elucidate the real mechanism of action of chia ingredients in the lowering of GI. Laparra and Haros [67] observed that when female rats were fed with a bread formulation containing 5% of chia seed the release of glucose in the blood was slow. Consumption of this type of bread may be beneficial in controlling the weight of obese people and it could help to prevent dysfunction in glucose metabolism.

**Table 5:** Effect of chia by-products in bread formulation on in vitro glycemic index estimation ^a^.

Formulation		Total Starch (%)	TSH_90_ (%)	AUC	GI	GL
Chia Ingredient	Level (%)					
Control (W)	0	79.1 ± 1.1 g	56.9 ± 5.5 c	5013	100 ± 2 d	57.0 ± 7.1 e
Chia Seeds (CWS)	5	74.5 ± 0.8 de	57.2 ± 0.0 c	4720	91.6 ± 2.0 c	52.4 ± 1.6 de
	10	71.7 ± 0.6 ab	48.1 ± 0.4 ab	3712	80.5 ± 3.1 ab	38.7 ± 1.7 abc
Whole Chia Flour (CWF)	5	75.6 ± 0.5 ef	48.4 ± 0.2 a	4228	86.1 ± 1.8 bc	41.4 ± 1.5 cd
	10	72.6 ± 0.0 b	42.1 ± 2.8 a	3215	75.0 ± 1.8 a	31.5 ± 1.0 ab
Semi-Defatted Chia Flour (CSDF)	5	74.2 ± 0.4 cd	51.5 ± 0.8 bc	4408	88.4 ± 2.5 c	45.5 ± 2.8 de
	10	70.7 ± 0.3 a	45.0 ± 3.0 ab	3293	77.0 ± 1.5 a	34.7 ± 4.2 abc
Low-Fat Chia Flour (CLFF)	5	76.4 ± 0.2 f	49.2 ± 0.9 abc	4308	87.3 ± 3.0 bc	42.9 ± 1.3 bcd
	10	72.9 ± 0.7 bc	43.8 ± 3.0 ab	3213	76.3 ± 3.0 a	29.9 ± 5.5 a

^a^ Mean ± standard deviation, *N* = 3. Values followed by the same letter in the same column are not significantly different at 95% confidence level. TSH_90_, Total starch hydrolyzed at 90 min; AUC, area under the curve of starch digestion; GI, glycemic index; GL glycemic load.

## 4. Conclusions

The incorporation of chia seeds or chia flour increased the nutritional value of bread products with regard to the concentrations of proteins with higher biological value, lipids with a higher proportion of omega fatty acids, and minerals compared to the control sample. It is important to emphasize that chia seeds and chia flour contain a high concentration of the basic amino acid lysine, which is an essential amino acid from a nutritional standpoint and deficient in cereals. Consequently, chia ingredients are beneficial in cereal products. The higher linolenic acid content of the samples containing chia seeds, due to the protection that the integrity of the seed cover provides against oxidation during baking, should be taken into account when formulating baked foods enriched with omega-3; encapsulation of the oil is imperative if fortification is required. However, the chia seeds and chia flour provided a better ω-3/ω-6 ratio according to the recommendations of WHO/FAO and EFSA, despite the loss of unsaturated acids in the oven. The contribution of iron was deficient, whereas the contributions of calcium and zinc were higher than in wheat bread, taking into account the fact that their bioavailability was conditioned by the molar ratio (Ins*P*_6_/mineral). The CSDFB10 and CLFFB10 breads could be included in the daily diet to control obesity and to prevent constipation owing to their low fat content and high amount of dietary fiber.

The breads with chia provide higher contributions to intake of calcium, iron, and zinc than wheat bread. Accordingly, bread with chia seed or chia flour meets almost all the daily requirement of these minerals in women and men, which is not the case with wheat bread. The bread with 10% chia could be used in a weight control diet because of its low glycemic load.

In the light of the present data, chia seed and chia flour could be used as a partial replacement of wheat flour in bread formulations, increasing the nutritional and functional value of the products, with important implications that could help to enhance their role in the prevention of metabolic diseases.

## Chemical Compounds Studied in This Article

L-histidine (PubChem CID: 6274); L-threonine (PubChem CID: 6288); L-tyrosine (PubChem CID: 6057); L-valine (PubChem CID: 6287); L-methionine (PubChem CID: 6137); L- phenylalanine (PubChem CID: 6140); L-tryptophan (PubChem CID: 6305); I-isoleucine (PubChem CID: 6306); L-leucine (PubChem CID: 6106); Lysine (PubChem CID: 5962); L-aspartic acid (PubChem CID: 5960); L-asparagine (PubChem CID: 6267); Glutamic acid (PubChem CID: 33032); L-glutamine (PubChem CID: 5961); Serine (PubChem CID: 5951); Glycine (PubChem CID: 750); L-arginine (PubChem CID: 6322); L-alanine (PubChem CID: 5950); L-proline (PubChem CID: 145742); L-Cysteine (PubChem CID: 5862); Palmitic acid (PubChem CID: 985); Stearic acid (PubChem CID: 5281); Arachidic acid (PubChem CID: 10467); Behenic acid (PubChem CID: 8215); Elaidic acid (PubChem CID: 637517); Oleic acid (PubChem CID: 445639); Linoleic acid (PubChem CID: 5280450); alpha-Linolenic acid (PubChem CID: 5280934); Phytic acid (PubChem CID: 890); *Myo*-inositol (PubChem CID: 892); D-Glucose (PubChem CID: 5793).

## Abbreviations

American Association of Cereal Chemists (AACC); Adequate intake (AI); Amino acid score (AAS); Alpha-linolenic acid (ALA); Association of Official Analytical Chemists (AOAC); Chia seeds (CWS); Chia whole flour (CWF); Control bread (WB); Cysteine (Cys); Dietary reference intakes (DRIs); dry matter (d.m.); Essential amino acids (EAAs); European Food Safety Authority (EFSA); European Union (EU); Females (F); Food and Agriculture Organization (FAO); Gas chromatography (GC); Glycemic index (GI); Glycemic load (GL); High Performance Liquid Chromatography (HPLC); Histidine (His); Hydrolysis index (HI); inositol tetrakisphosphate (Ins*P*_4_); inositol trisphosphate (Ins*P*_3_); inositol pentakisphosphate (Ins*P*_5_); Isoleucine (Ile); Linoleic acid (LA); Low-fat chia flour (CLFF); Low-Fat chia flour bread, replaced at 5% (CLFFB5); Low-fat chia flour bread, replaced at 10% (CLFFB10); Lysine (Lys); Males (M); Methionine (Met); Metionine+Cysteine (Met+Cys); Monounsaturated acid (MUFA); Non-essential amino acid (NEAA); Phenylalanine+Tyrosine (Phe+Tyr); Phytic acid (Ins*P*_6_); Polyunsaturated fatty acids (PUFAs); Popular reference intake (PRI); Recommended dietary allowance (RDA); Saturated fatty acids (SFA); Semi-defatted chia flour (CSDF); Semi-defatted flour bread replaced at 5% (CSDFB5); Semi-defatted flour bread replaced at 10% (CSDFB10); Total dietary fiber (TDF); Tryptophan (Trp); Tyrosine (Tyr); United Nations University (UNU); United States Department of Agriculture (USDA); Valine (Val); Wheat flour (W); Whole seed bread, replaced at 5% (CWSB5); Whole Seed Bread, replaced at 10% (CWSB10); Whole flour bread, replaced at 5% (CWFB5); Whole flour bread, replaced at 10% (CWFB10); World Health Organization (WHO).
